# Short term modulation of trunk neuromuscular responses following spinal manipulation: a control group study

**DOI:** 10.1186/1471-2474-14-92

**Published:** 2013-03-13

**Authors:** Marie-Pierre Harvey, Martin Descarreaux

**Affiliations:** 1 Département des sciences de l’activité physique, Université du Québec à Trois-Rivières, Trois-Rivières, G9A 5H7, Canada; 2 Département de chiropratique, Université du Québec à Trois-Rivières, Trois-Rivières, G9A 5H7, Canada

**Keywords:** Spinal manipulation, Electromyography, Kinematics, Flexion-relaxation phenomenon

## Abstract

**Background:**

Low back pain (LBP) is one of the most frequent musculoskeletal conditions in industrialized countries and its economic impact is important. Spinal manipulation therapy (SMT) is believed to be a valid approach in the treatment of both acute and chronic LBP. It has also been shown that SMT can modulate the electromyographic (EMG) activity of the paraspinal muscle. The purpose of this study was to investigate, in a group of patients with low back pain, the persistence of changes observed in trunk neuromuscular responses after a spinal manipulation (SMT).

**Methods:**

Sixty adult participants with LBP performed a block of 5 flexion-extension movements. Participants in the experimental group (n=30) received lumbar SMT whereas participants in the control group (n=30) were positioned similarly for the treatment but did not receive SMT. Blocks of flexion-extension movements were repeated immediately after the manipulation as well as 5 and 30 minutes after SMT (or control position). EMG activity of paraspinal muscles was recorded at L2 and L5 level and kinematic data were collected to evaluate the lumbo-pelvic kinematics. Pain intensity was noted after each block. Normalized EMG, pain intensity and lumbo-pelvic kinematics were compared across experimental conditions.

**Results:**

Participants from the control group showed a significant increase in EMG activity during the last block (30 min) of flexion-extension trials in both flexion and full-flexion phases at L2. Increase in VAS scores was also observed in the last 2 blocks (5 min and 30 min) in the control group. No significant group x time interaction was seen at L5. No significant difference was observed in the lumbo-pelvic kinematics.

**Conclusion:**

Changes in trunk neuromuscular control following HVLA spinal manipulation may reduce sensitization or muscle fatigue effects related to repetitive movement. Future studies should investigate short term changes in neuromuscular components, tissue properties and clinical outcomes.

## Background

Low back pain (LBP) is one of the leading cause of activity limitation and work absence in western countries, consequently raising important social and economic challenges [1]. Dunn et al. [2] estimated that 1 out of 5 adults is affected by LBP, whereas 40% of the population has experienced symptoms during the previous month [2]. According to various epidemiological studies, approximately 58-80% of the population will experience an episode of LBP at least 1 time in their life [1,3-5]. Most cases (around 85%) are classified as non-specific because no definitive pathology can be associated with the low back pain condition [6].Qualified by some authors as an epidemic, this affection is one of the most common reasons for medical consultation [7]. Dagenais, Caro, & Haldeman [8] estimated the total cost related to low back pain in United-States to be in the range of 84.1 to 624.8 billion dollars per year, including direct and indirect costs [8].

Among conservative approaches, manual therapy, including spinal manipulation therapy (SMT) as well as mobilization, has been suggested to be an appropriated therapeutic option in the treatment of both acute and chronic LBP [7,9]. However, the reported effect size and clinical improvement are modest and the “active ingredient” underlying clinical improvement remains unclear.

The physiological mechanisms underlying SMT related clinical improvements remain to be determined. Among the possible explanations, biomechanical changes have been hypothesized as possible factors involved in clinical responses to SMT. SMT may release meniscoids or adhesions in the joint, reduce distortion on the intervertebral disc and reduce mechanical stress or strain in soft and hard spinal tissues [10]. Changes in neurophysiological responses have also been suggested as possible mechanisms underlying clinical effects. In 2012, a review by Haavik and Murphy suggested a central mechanism of action for SMT (central processing of proprioceptive afferent input) [11]. Under such hypothesis, SMT would lead to plastic changes in sensorimotor integration within the central nervous system [11]. Moreover, neurophysiological responses to SMT can be illustrated by the high frequency discharge observed in primary afferents paravertebral neurons that occurs immediately after the SMT [10].

Early work investigating physiological responses to SMT in healthy participants also revealed the presence of an electromyographic (EMG) response in paraspinal and limb muscles following SMT [12,13]. Similar surface EMG studies were also conducted in various clinical populations. De Vocht and Pickar observed a 25% reduction of paraspinal muscles EMG activity in a group of patients with low back pain who received SMT [14]. Similar immediate SMT effects were also reported when functional responses (flexion relaxation phenomenon) were evaluated during a flexion-extension task [14-16]. Although preliminary evidence suggests that exercises alone or in combination with spinal manipulation (over an 8-week period) can modulate trunk neuromuscular response in people with chronic neck pain, the independent contribution of spinal manipulation and the persistence in these changes remain to be determined [17].

Indahl et al. [18] observed, in an animal model (domestic pigs), a reduction of multifidus and longissimus muscles EMG activity at L4-L5 in response to electrically-induced pain following zygapophyseal joint capsule distension [18]. Such decrease in EMG activity was observed following the injection of a physiological saline solution in the zygapophyseal. EMG activity decreased following capsular distension and such effects was observed over a period of 30 minutes (when the experiment was stopped). Following these results, the authors suggested that the inhibitory discharges from the zygapophyseal joint capsule may explain the clinical results obtained with manipulative treatment and mobilization of the zygapophysial joints [18].

To our knowledge, persistence of neuromuscular changes following SM has never been investigated in subjects with chronic non-specific low back pain. Consequently, the main objective of this study was to investigate the nature and duration of EMG and kinematic changes triggered by SM in this population. A secondary objective of the study was to assess changes in pain associated with the intervention and task repetition.

## Methods

### Participant

Sixty participants (26 men and 34 women) with low back pain were included in this study and randomly assigned to either the experimental or the control group. To be included in study, participants had to be diagnosed with non-specific low back pain (mechanical origin). Participants were excluded if they presented with any of the following conditions: inflammatory rheumatic disease, infectious disease, neuromuscular disease, vascular disease, connective tissue disease, severe disabling pain, morbid obesity, neurologic signs and symptoms and pregnancy. All participants gave their written informed consent. Ethical approval for the study was granted by the Université du Québec à Trois-Rivières ethics committee (Ref. No. CER-10-156-06.07). Prior to the experimentation, each participant underwent a brief clinical evaluation to confirm their clinical status (non-specific low back pain) and to determine the presence of any contraindication to spinal manipulative therapy (if so the participant was excluded). They then completed the following questionnaires: the modified Oswestry disability index questionnaire (ODI), the fear avoidance belief questionnaire (FABQ), and visual analog pain scale (VAS: 100 mm from no pain to worst possible pain). Using a VAS score (100 mm from no pain to worst possible pain), pain was also assessed after each set of 5 flexion-extension movements. Baseline characteristics of participants are presented in Table 1.

**Table 1:** Participants’ baseline characteristics

	**Experimental group (n=30)**		**Control group (n=30)**		**p value**
Age (y)	31.3	± 11.2	34.3	± 12.4	0.42
Height (cm)	171.6	± 7.9	174.9	± 8.5	0.14
Weight (Kg)	73.3	± 13.0	77.8	± 13.7	0.20
mODI (/100)	19.8	± 11.4	16.9	± 12.5	0.36
FABQ-physical activity (/24)	10.4	± 6.3	10.7	± 6.4	0.97
FABQ work (/42)	10.6	± 7.9	12.1	± 10.0	0.42
VAS before (/100)	27.5	± 22.5	19.6	± 18.7	0.32
VAS after (/100)	33.8	± 26.0	31.3	± 26.3	0.72

Mean ± standard deviation.

### Procedures

#### Trunk flexion-extension tasks

The trunk flexion-extension task consisted of four movement phases: 1) The subject stands still for 3 s (Quiet standing); 2) The subject bends forward over 5 s to reach a fully-flexed position (Flexion); 3) The fully-flexed position is held for 3 s (Full flexion); and 4) Trunk extension enables the subject to return to the initial upright position over 5 s (Extension). Movement was paced using an auditory metronome and verbal instructions were given to standardize the task. Five successive flexion-extension movements were performed by each participant before and immediately after a spinal manipulation (or control mobilization) applied to the middle lumbar segment.

The participants from the experimental group (n=30; 16 men and 14 women) were asked to lie down on the chiropractic table on their left side. Their trunk was slightly rotated to the right, with arms crossed over the chest. The left lower limb was extended, whereas the right leg and thigh were flexed at a 90° angle. An experienced clinician (20 years of practice as a chiropractor), blinded to the study objectives and experimental conditions, faced the participants at approximately 45°, stabilizing the subjects' right leg between the thighs and the trunk with his right hand. The chiropractor's fingers (left hand) made contact with the lateral margin of the L3 spinous process, and an impulse thrust with a lateral to medial vector was applied to the vertebral segment. This procedure has been described as a lumbar spinous pull by Peterson and Bergman [19]. The procedure as well as the targeted spinal segment (L3) were chosen mainly for technical purposes, namely to avoid any displacement of data acquisition instrumentation. Participants from the control group (n = 30; 18 men and 12 women) were positioned in a same left-side–lying posture, with the superior knee flexed and the trunk slightly rotated for 5 seconds. No spinal manipulation, however, was given. Subsequently, two sets of flexion-extension movements were performed 5 and 30 minutes after the manipulation.

### Measurements

#### Electromyography

Surface electromyography (sEMG) data were collected using bipolar electrodes applied bilaterally over the lumbar erector spinae muscles at the L2-L3 level and at the L4-L5 level (~3 cm from the midline). A ground electrode was placed over the left anterior superior iliac spine. Usual measures were taken to improve skin impedance: excessive hair shaving, slight skin abrading with sandpaper and cleaning of skin with alcohol. EMG activity was recorded using a Delsys EMG sensor (Model DE2.1, Delsys Inc., Boston, MA, USA) with a common mode rejection ratio of 92 dB at 60 Hz, an input impedance of 10^15^ Ω, and analog to digital converted at 1000 Hz with a 12-bit A/D converter (PCI 6024E, National Instruments, Austin, TX, USA). EMG data were filtered digitally by a 10 to 450 Hz bandpass, zero-lag and fourth-order Butterworth filter. Data were collected by Labview (National Instruments, Austin, TX, USA) and processed by Matlab (MathWorks, Natick, MA, USA).

**Figure 1:**
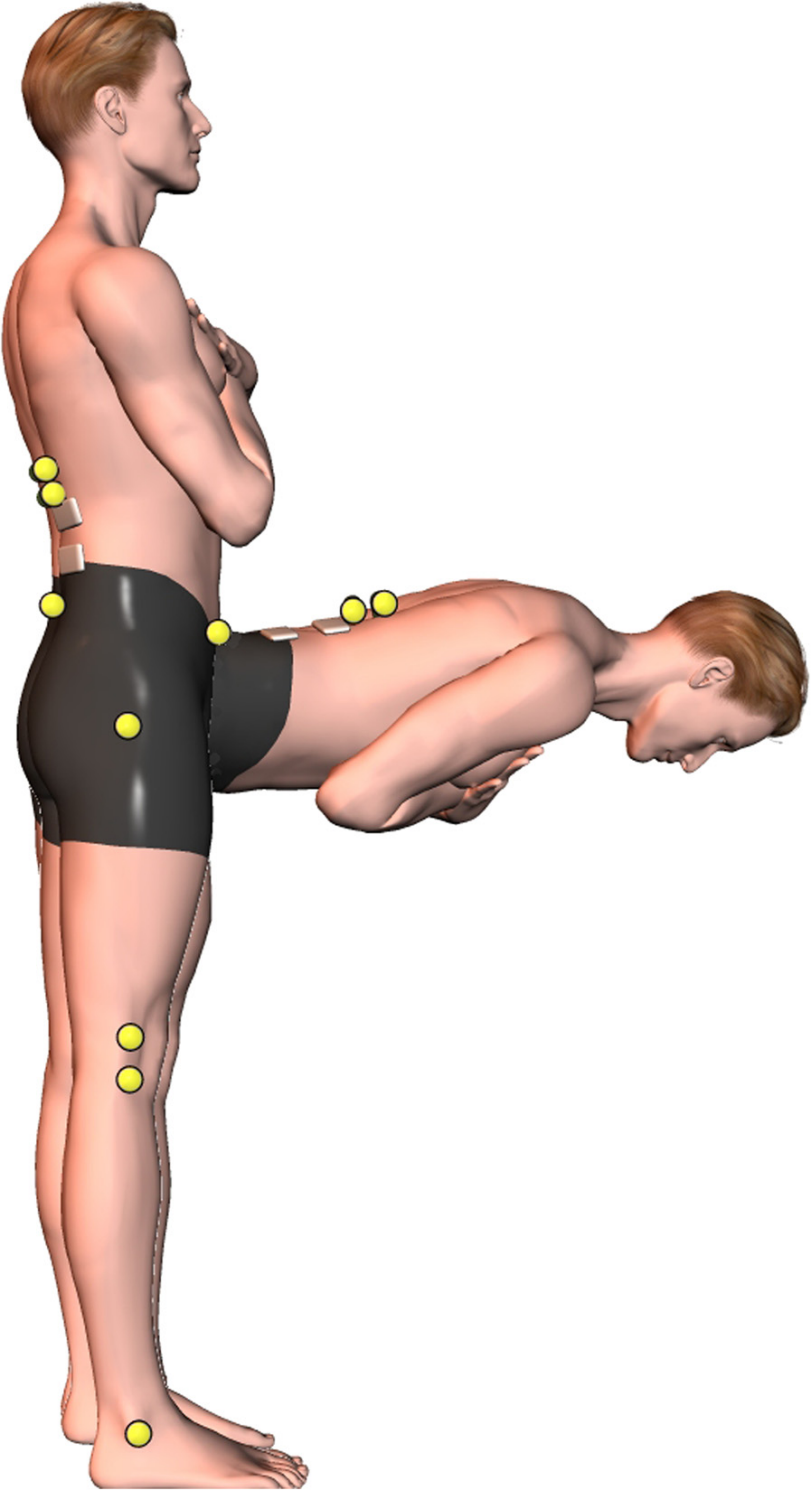
Representation of the experimental setup, including 8 infrared LEDs and EMG electrodes at L2 and L5 (erector spinae).

The root mean square (RMS) of the sEMG signals was calculated for each of the four phases of the flexion-extension task. RMS values were normalized using the RMS value in the extension phase of the first pre-intervention trial [20]. Left and right normalized EMG values were compared using Student’s t-tests. Since no difference was observed (p>0.05), left and right EMG data were averaged for each segment (L2-L3 and L4-L5) [20,21]. The experimental setup is presented in Figure 1.

#### Kinematics

Kinematics data were collected by a motion analysis system (OptotrakCertus, Northern Digital Inc., Waterloo, ON, Canada). Light-emitting diodes (LED) were positioned on the right side and back of the participant on 8 anatomical landmarks: (a) external malleolus; (b) Gerdy's tubercle; (c) lateral condyle of the femur; (d) greater trochanter; (e) anterior superior iliac spine (ASIS); (f) posterior superior iliac spine (PSIS); (g) L1; (h) T11. Data were sampled at 100 Hz and low-pass filtered by a dual-pass, fourth-order Butterworth filter with a cutoff frequency at 5 Hz.

Raw kinematic data were transformed into angles to evaluate the movement of the hip and the lumbar regions. Each angle was created by two converging vectors, each of them resulting from a line drawn between two LEDs. The hip angle was formed from the pelvic plateau vector (ASIS - PSIS) and the thigh vector (lateral condyle of the femur - greater trochanter). The lumbar angle resulted from the combination of the dorsal vector (T11 - L1) and the pelvic plateau vector (ASIS - PSIS). The lumbar and hip angles served to calculate the lumbar to hip (L/H) ratio which reports the specific contribution of both lumbar region and hip articulation to the movement. Total trunk flexion and extension angles were both divided in quartiles (Q1-Q4) for which the L/H ratio was computed [20-22]. The experimental setup is presented in Figure 1.

### Statistical analyses

Normality of distribution for every dependent variable was assessed with the Kolmogorov-Smirnov test and through visual inspection of data. All data were analysed according to a pre-established experimental design using Statistica software version 10 (StatSoft, Tulsa, OK, USA). One-way ANOVA was performed. T-tests for dependent samples were conducted for baseline values of continuous variables. Two-way (Group X Time) repeated-measures analyses of variance (ANOVAs) were conducted for each dependent variable (EMG, kinematics and pain). Since baseline analyses revealed a significant difference in baseline VAS scores, data were also analyzed using ANCOVAs, where group and time intervals represented the main factors and VAS scores the continuous predictor. Finally, whenever ANOVAs yielded a significant time effect for the VAS scores, polynomial contrasts were conducted to test for the linear trend. Statistical significance for all analyses was set at p < 0.05 (2-tailed).

## Results

Both groups were comparable (see Table 1) for age, weight, height, disability index and fear avoidance belief scores (all p>0.05). A significant difference in baseline pain scores (VAS) was observed between the two groups (p=0.028).

Normalized RMS values during quiet standing, flexion, full flexion and extension phases were compared between groups and across all time intervals. Repeated-measures ANOVA yielded a main effect of time during the following movements phases: quiet standing at L2 (F(3,165)=3.1442, p=0.02), flexion-relaxation at L2 (F(3,165)=6.0123, p<0.001) and L5 (F(3,165)=2.8121, P=0.04) and extension at L2 (F(3,165)=6.2103, p<0.001). Table 2 presents the mean normalized RMS values for both groups during each phases of movement at each time of experimentation.

**Table 2:** Mean ± standard deviation normalized RMS values for both groups during each movement phase throughout the experimentation

**Phases**	**Level**	**Time**	**Experimental group (n=30)**		**Control group (n=30)**	
Quiet Standing	L2	Baseline	0.367	± 0.120	0.367	± 0.116
		Post SMT	0.379	± 0.125	0.371	± 0.122
		Post SMT 5 min	0.367	± 0.131	0.362	± 0.121
		Post SMT 30 min	0.376	± 0.125	0.400	± 0.124
	L5	Baseline	0.321	± 0.115	0.340	± 0.153
		Post SMT	0.319	± 0.108	0.352	± 0.161
		Post SMT 5 min	0.313	± 0.114	0.344	± 0.162
		Post SMT 30 min	0.321	± 0.134	0.356	± 0.183
Flexion	L2	Baseline	0.577	± 0.190	0.587	± 0.168
		Post SMT	0.558	± 0.193	0.575	± 0.185
		Post SMT 5 min	0.560	± 0.171	0.573	± 0.163
		Post SMT 30 min	0.554	± 0.179	0.620	± 0.183
	L5	Baseline	0.601	± 0.150	0.578	± 0.151
		Post SMT	0.559	± 0.119	0.589	± 0.172
		Post SMT 5 min	0.584	± 0.162	0.617	± 0.249
		Post SMT 30 min	0.590	± 0.141	0.590	± 0.166
Full flexion	L2	Baseline	0.413	± 0.256	0.477	± 0.251
		Post SMT	0.410	± 0.271	0.449	± 0.267
		Post SMT 5 min	0.377	± 0.252	0.434	± 0.228
		Post SMT 30 min	0.393	± 0.254	0.513	± 0.276
	L5	Baseline	0.423	± 0.261	0.428	± 0.278
		Post SMT	0.376	± 0.238	0.406	± 0.277
		Post SMT 5 min	0.377	± 0.246	0.403	± 0.272
		Post SMT 30 min	0.412	± 0.252	0.449	± 0.263
Extension	L2	Baseline	0.986	± 0.056	1.003	± 0.085
		Post SMT	0.975	± 0.097	0.991	± 0.130
		Post SMT 5 min	0.977	± 0.099	0.990	± 0.125
		Post SMT 30 min	1.005	± 0.116	1.040	± 0.180
	L5	Baseline	0.995	± 0.062	0.997	± 0.094
		Post SMT	0.940	± 0.092	1.009	± 0.180
		Post SMT 5 min	0.940	± 0.100	1.004	± 0.181
		Post SMT 30 min	0.974	± 0.14	0.99	± 0.180

The analysis also revealed significant group x time interactions for the flexion (F(3,165)=3.5487, p=0.016) and full flexion phases (F(3,165)=4.5796, p<0.001) of movement at L2. Post hoc analysis indicated that the control group, for both variables, showed a significant increase in EMG activity during the last block (30 min) of flexion-extension trials (Tukey’s test; p<0.001). No significant (p>0.05) group, condition or interaction effect was observed at the L5 level. ANCOVAs (analyses adjusted for baseline pain scores) yielded results similar to those obtained with the initially planned ANOVAs for all EMG and kinematics variables indicating that differences in baseline pain scores cannot explain the observed differences.

**Figure 2:**
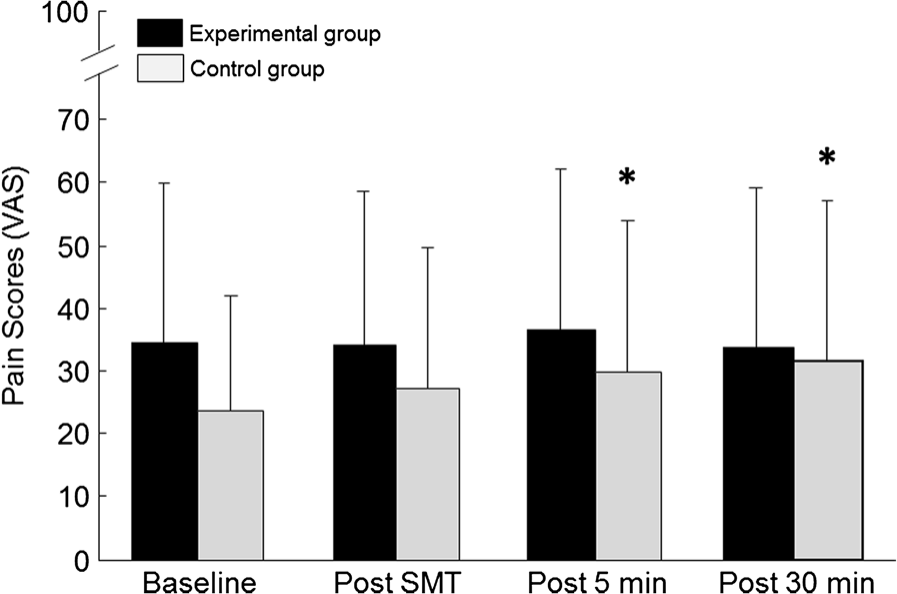
**Mean baseline and post spinal manipulation pain scores (VAS = 0–100) for both the control and experimental groups.** *Pain in the control group significantly increased at the 5 min and 30 assessments when compared to baseline value. Whiskers indicate standard deviation.

**Figure 3:**
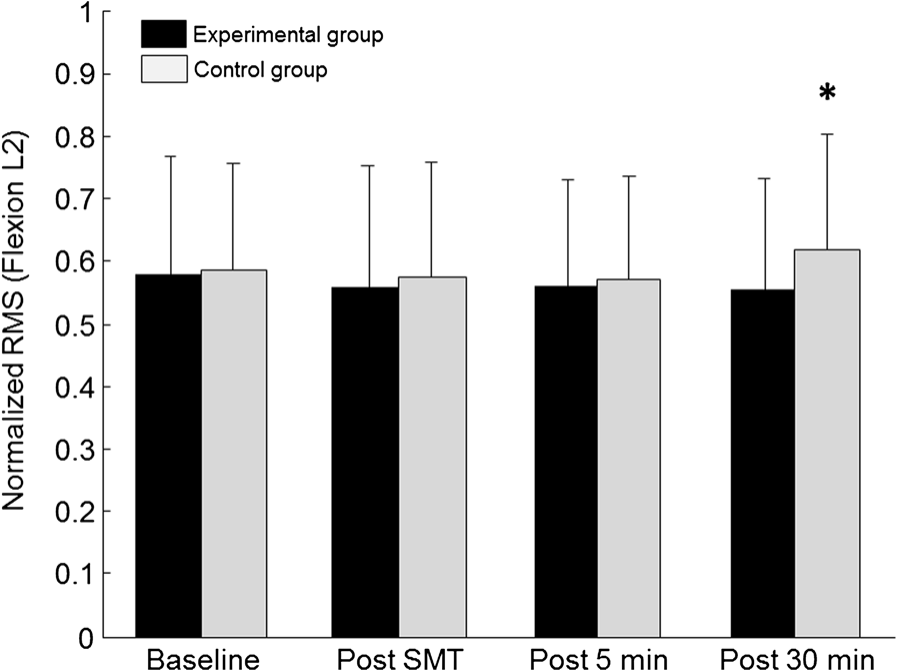
**Mean baseline and post spinal manipulation L2 paraspinal normalized RMS values (EMG) for both the control and experimental groups during the flexion phase of the task.** *RMS values in the control group significantly increased during the last block of trials. Whiskers indicate standard deviation.

**Figure 4:**
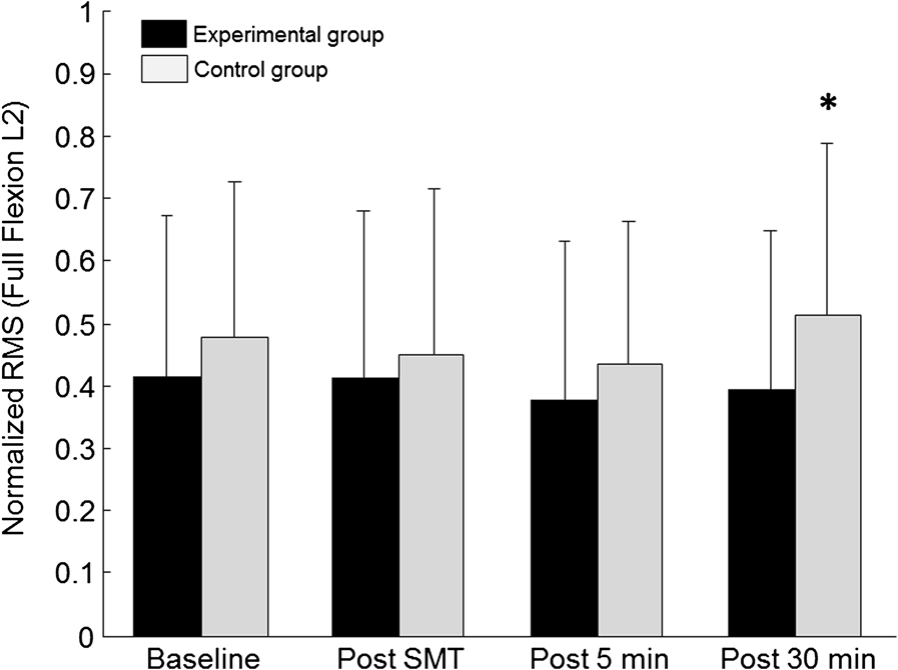
**Mean baseline and post spinal manipulation L2 paraspinal normalized RMS values (EMG) for both the control and experimental groups during the full flexion phase of the task.** *RMS values in the control group significantly increased during the last block of trials. Whiskers indicate standard deviation.

A significant interaction was also observed for VAS scores which showed a significant increase in the control group during the last two blocks (5 min and 30 min) of flexion-extension trials (Tukey’s test; p<0.01) and polynomial contrasts confirmed the linear increase in pain overtime (p<0.01). Figures 2, 3 and 4 respectively illustrate pain scores and paraspinal muscles EMG activity throughout the experiment (L2 during flexion and full flexion).

Trunk and hip flexion angles were obtained to calculate the L/H ratio and are used to assess the overall kinematics as well as the movement strategy during trunk flexion across the various conditions. All quartiles of L/H ratios were also compared between groups and across all time intervals according to the experimental design and the analyses. The analyses did not reveal any significant main or interaction effects for the various L\H ratios (all ps >0.05).

## Discussion

The aim of the present study was to determine the effect of SM on the EMG activity of the paraspinal muscles and the duration of such effect over a 30 min period of time.

Interestingly, results showed that EMG activity at L2 increased only in the control group after 30 minutes. A gradual increase in the VAS scores was also observed in the same group over the 30 minute period.

The present results partly differ from previous studies. Using the same experimental paradigm, Lalanne et al. [15] observed a decrease in EMG activity at the L2 level immediately following a lumbar SM at L3 level [15].Similar results were also reported by Bicalho at al. [16] who showed decreases in EMG activity at the L5-S1 level following a SM at L4-L5 segment [16]. Such results have not been reproduced in the present study. The changes observed in the above mentioned studies, however, indicated that EMG responses were mostly segmental (changes observed only at the contacted or adjacent spinal segment) [15,16]. Despite the fact that decreases in EMG responses immediately following SMT were not observed in the present study, significant group differences observed during at the 30 min assessment were present for the L2 segment (SMT was performed at L3) whereas changes were not observed at L5. Interestingly, these changes were not associated with changes in lumbo-pelvic kinematics. In a recent review, Millan et al. [23] reported that none of the selected studies of the lumbar spine showed an immediate effect of SMT on lumbar range of motions. Future studies should include assessment for an extended period of time (hours and days) in order to better document the association between neuromuscular response to SMT and changes in lumbo-pelvic kinematics.

The combination of increased paraspinal EMG activity and increased pain observed in the control group during the last block of trials, although unexpected, raises important questions regarding the possible effects of SMT. These results suggest that a trial-to-trial “sensitization effect”, observed in the control and leading to increased paraspinal muscle activity, did not occur in the SMT group. In a recent review of literature, Millan et al. [24] explored the short term effect of SM following experimentally induced pain. The review suggested both a local and regional effect of SM on pain reduction. The outcome of SM was also affected by the method of pain induction as pain induced by pressure, electricity, stretching of painful tissue, dermal irritation and spontaneous pain all responded to SMT. Such results were not observed, however, for temperature-induced pain. The specific effect of SMT on sensitization phenomenon should be further investigated in future studies.

Alternatively, changes in trunk muscle activity may also been explained by changes in paraspinal tissue properties. Olson et al. [25] showed increased paraspinal muscle EMG during the flexion following cyclic flexion extension exercise over 9 minutes [25]. These changes were accompanied by random EMG activity (described by the authors as spasms). Changes observed in the present study may therefore result from both modifications in spinal tissues properties and muscle fatigue. Moreover, sustained flexed or semi-flexed spinal sitting postures may result in increased paraspinal muscle activity [26] and provocation or aggravation of existing pain [26,27]. Therefore, increases in VAS scores and EMG activity following the 25 minutes of “sitting posture” may have been triggered by changes in paraspinal tissues caused by static lumbar flexion loading. Specific mechanisms underlying between group differences during flexion and full flexion and the potential role of SMT remain to be investigated.

### Study limitations

As for all manual therapies, true blinding of participants was impossible during the experimentation. Participant’s expectations towards receiving (or not receiving) spinal manipulation may have affected the VAS scores, but one could argue that it is less likely to affect EMG activity. Besides, because spinal manipulations were delivered by a clinician, no standardization of the force and speed parameters was possible, potentially inducing a bias in the physiological response to SMT. According to Kawchuk et al. [28], a typical clinician’s trial-to-trial variability can reach 37 N when a peak force of 253 Newtons is used [28]. Finally, it was decided, mainly for technical reasons, that all SMTs would be delivered to the same spinal segment (L3), regardless of pain localization. Because pain and EMG responses overtime seem to follow similar patterns, delivering spinal manipulation according to pain patterns may have yielded different results. SMT procedures and delivering forces at the same segment for all subjects may not reflect the usual clinical practice where a specific joint will be targeted according to manual palpation and other clinical findings. It is therefore possible that the changes observed in the present study may not reflect exactly those encountered in a clinical setting.

### Clinical implications

Assessing the clinical relevance of EMG changes following SMT remains challenging. However, the changes reported in this study (as high as 10-15% in normalized RMS values) may be viewed as significant changes in erector spinae recruitment during a typical activity of daily living (flexing the trunk). Such changes, repeated over time may lead to muscle fatigue and changes in spinal stability.

A recent review by Millan and al. [24] suggested that SMT has a hypoalgesic effect both locally (segmental level only) and regionally (related to the segmental innervation). The present results, although preliminary, suggest a possible modulation of sensitization phenomenon observed in chronic low-back pain populations [29]. A recent study suggested that descending pain modulation may shift from descending inhibition towards descending facilitation following repetitive muscle contractions in chronic pain populations [30]. SMT may have, for a brief period of time (30 minutes), limited the effect of muscle fatigue on pain processing mechanisms. The exact nature and extend (magnitude) of these effects are unclear and future study regarding the SMT in presence of muscle fatigue and changes in a tissue properties should be considered.

## Conclusion

The present results indicate that changes in trunk neuromuscular control following HVLA spinal manipulation may reduce sensitization or muscle fatigue effects related to repetitive movement. Future studies investigating short term changes in neuromuscular components, tissue properties and clinical outcomes should integrate repeated assessments over time to better evaluate the clinical relevance of these changes.

## Competing interests

Authors declare they have no conflicts of interest. This study was funded through the Chaire de recherche en chiropratique FRCQ, The Fonds de recherche du Québec-Santé and the Canadian Chiropractic Association. The funding sources had no role in study design, data collection, analysis, data interpretation, or writing of the manuscript.

## Authors’ contributions

MD contributed to trial design and protocol development, had overall responsibility for the conduct of the study, and contributed to the experimentation, data analysis, writing of the manuscript and supervision of MPH. MPH, as part of her master’s degree thesis, conducted all experimental sessions, statistical analysis, and manuscript preparation. Both authors read and approved the final manuscript.

## Pre-publication history

The pre-publication history for this paper can be accessed here:

http://www.biomedcentral.com/1471-2474/14/92/prepub
